# Conformational plasticity of RepB, the replication initiator protein of promiscuous streptococcal plasmid pMV158

**DOI:** 10.1038/srep20915

**Published:** 2016-02-15

**Authors:** D. Roeland Boer, José Angel Ruiz-Masó, Manuel Rueda, Maxim V. Petoukhov, Cristina Machón, Dmitri I. Svergun, Modesto Orozco, Gloria del Solar, Miquel Coll

**Affiliations:** 1 Institute for Research in Biomedicine (IRB Barcelona), Barcelona, 08028 Spain; 2 Institut de Biologia Molecular de Barcelona (Consejo Superior de Investigaciones Científicas), Barcelona, 08028, Spain; 3 Centro de Investigaciones Biológicas (Consejo Superior de Investigaciones Científicas), Madrid, 28040, Spain; 4 European Molecular Biology Laboratory, Hamburg Unit, EMBL c/o DESY, Hamburg, 22607, Germany; 5 Departament de Bioquímica, Facultat de Biologia, Universitat de Barcelona, Barcelona, 08028, Spain

## Abstract

DNA replication initiation is a vital and tightly regulated step in all replicons and requires an initiator factor that specifically recognizes the DNA replication origin and starts replication. RepB from the promiscuous streptococcal plasmid pMV158 is a hexameric ring protein evolutionary related to viral initiators. Here we explore the conformational plasticity of the RepB hexamer by i) SAXS, ii) sedimentation experiments, iii) molecular simulations and iv) X-ray crystallography. Combining these techniques, we derive an estimate of the conformational ensemble in solution showing that the C-terminal oligomerisation domains of the protein form a rigid cylindrical scaffold to which the N-terminal DNA-binding/catalytic domains are attached as highly flexible appendages, featuring multiple orientations. In addition, we show that the hinge region connecting both domains plays a pivotal role in the observed plasticity. Sequence comparisons and a literature survey show that this hinge region could exists in other initiators, suggesting that it is a common, crucial structural element for DNA binding and manipulation.

RepB is the rolling-circle replication (RCR) initiator protein encoded by the 5541-bp promiscuous plasmid pMV158, originally isolated from *Streptococcus agalactiae* and involved in antibiotic resistance spread. It provides both an endonuclease function that constitutes the first step of RCR, and a strand-transfer activity that, together with the endonuclease activity, catalyses the replication termination step^1^. RepB is a 24 kDa polypeptide that purified and crystallised as a hexamer^2^,^3^. The DNA-binding capability as well as the nuclease and strand-transfer activities of RepB reside in its N-terminal origin binding domain (OBD), which belongs to the replication (Rep) class of the HUH endonuclease superfamily. HUH endonucleases are widespread in all three domains of life, where they perform numerous functions by catalysing cleavage and rejoining of single-stranded DNA (reviewed in^4^). The endonuclease domains of the members of this superfamily share a similar structure and a common catalytic mechanism that uses one or two active-site Tyr residue(s) and a divalent metal ion coordinated by, among other ligands, the His pair of the His-hydrophobic-His (HUH) sequence motif that names this superfamily^2^,^4^,^5^. The Rep class of the HUH endonucleases includes RCR initiators of plasmids, bacteriophages and viruses^6^. The activity of the Rep proteins has been speculated to be involved in the original recombination events that generated the ancestors of two sequence similarity-based RecRep gene families^7^. RepB from pMV158 is related to the N-terminal half of the RecRep2 family-encoded proteins, which also contain a C-terminal region similar to the picorna-like virus 2C protein, assigned to the superfamily 3 (SF3) helicases^7^. RecRep2-like genes have been identified in plasmids from phytoplasmas^8^ and in the genomes of *Lactobacillus acidophilus*, *Lactococcus lactis* and *Phytoplasma asteris*^7^. A second RecRep1 family of hybrid proteins, consisting of an N-terminal part related to the Rep proteins of nanoviruses and a C-terminal part related to the 2C proteins of picorna-like viruses, is represented by the circovirus Rep initiators and could also be encoded by the genomes of the *Canarypox* virus, *Entamoeba histolytica* and *Giardia duodenalis*, and by plasmid p4M from *Bifidobacterium pseudocatenulatum*.

RepB is unique among plasmid replication initiators because it is purified as a hexamer, whereas other plasmid initiators are purified as monomers or dimers^9^,^10^. Hexamerisation of the initiator protein is, in contrast, common among small plant and animal viruses. The viral initiators also contain at their C-terminus an SF3 helicase domain^11^, which is missing in RepB. The structure of a truncated replication initiator E1 from bovine papillomavirus (BPV) shows that the protein contains an oligomerisation domain (OD) responsible for hexamerisation^12^. The structures of full-length RepB also revealed the presence of an OD of similar fold^2^. Although the existence of an OD in E1 and related proteins has already been noted^11^,^13^, a thorough analysis of the fold similarities of the OD from different atomic structures has not been reported.

RepB is to date the only published example of an atomic structure of a Rep protein from RCR plasmids or bacteriophages^2^,^4^, and the only Rep structure that includes both OBD and OD. Whereas the RepB ODs are arranged with C6 local symmetry, the N-terminal OBD domains are found in nine distinct orientations relative to the OD in the two different structures determined (Fig. 1). The OBDs show few preferred interactions with the ODs or with neighbouring OBDs and therefore do not appear to contribute to the RepB hexamer formation. The loosely-coupled domain arrangement in RepB_6_ allows a high level of conformational freedom of the OBDs that may be related to its role in generating an active replisome.

In addition to RepB, several viral OBDs in complex with DNA have been structurally characterized at atomic level, such as those of BPV E1 helicase^14^, adeno-associated virus 5 (AAV5) Rep^15^ and simian virus 40 large tumour antigen (SV40 LTag)^16^. These structures provide detailed information on interactions between the OBD and the DNA. An integrated analysis of the available structures of proteins with different domain compositions would aid in assessing the importance and implications of the OBD movement in the function of these hexameric replication initiators.

Here we confirm the existence of a RepB-like all-helical OD domain responsible for oligomerisation in viral Rep proteins and replication initiators from plasmids of the pMV158 family. We further confirm the preservation of the hexamerisation state of RepB in solution at different concentrations using SAXS. We show that *in silico* simulations reproduce and confirm the movement of the OBD inferred from the crystal structures. Separate expression and purification of RepB OD and OBD confirms that OD is essential for hexamerisation. We provide an experimental X-ray structure for the Ba^2+^-RepB_6_ complex and describe, using both experimental and theoretical techniques, the OBD conformational landscape in the RepB hexamer, showing that the protein displays large intrinsic flexibility at the OBDs level, allowing for fluctuations among multiple conformational states. We propose that the combination of an hexameric OD ring rigid scaffold and hinge-separated flexible additional domains, in particular the OBDs, is a common structural feature that enables hexameric initiators of the pMV158 plasmid family and of different animal and plant viruses to recognise the replication origin.

## Results and Discussion

**Figure 1:**
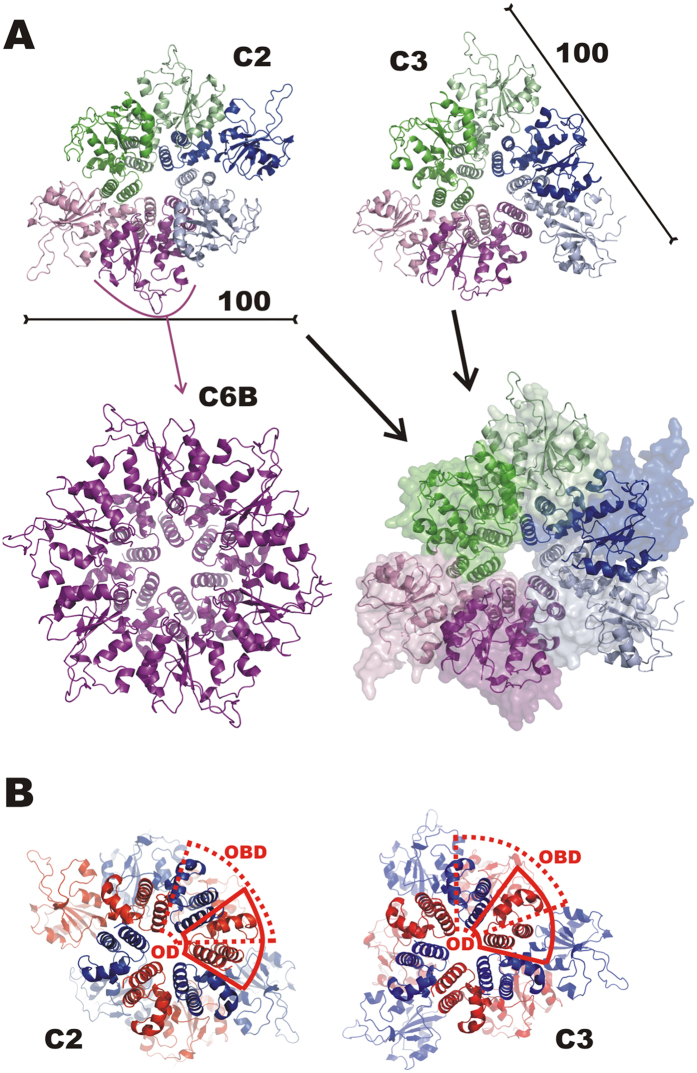
OBD conformations in the C2 and C3 crystal structures of RepB. (**A**) Views on the OBDs of the cartoon representations of the respective C2 and C3 crystal structures and after superposition using the ODs of both (C2 structure shown as a transparent surface in the superposition). The C2 protomer that is hexamerised to give the C6B model is indicated. The scale bars indicate the approximate dimension. (**B**) Views on the ODs of the cartoon representation of the C2 and C3 structures along the C6 axis of the ODs. The protomers are now alternately coloured red and blue, the dashed and continuous red outlines mark the boundaries of the OBD and OD, respectively, of one of the protomers. The figure shows that the OD of a blue-coloured neighbouring protomer is partly located above the OBD of a red-coloured protomer.

**Figure 2:**
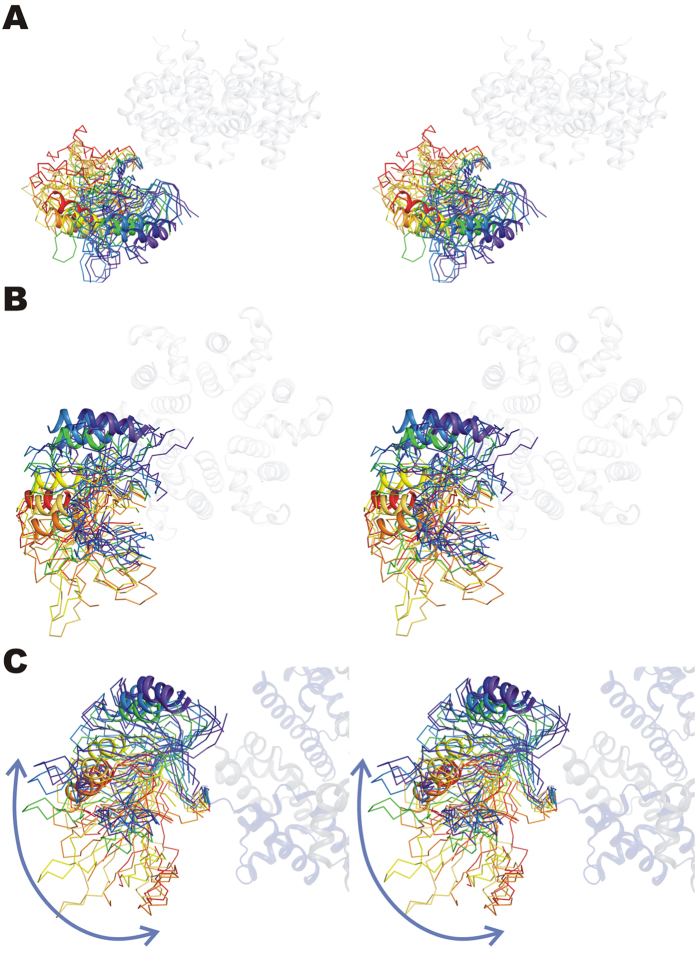
Superposition of the ODs of all nine protomers of the C2 and C3 structures showing the orientation of the OBDs with respect to the OD ring. The OD hexamer is shown in blue and the different OBDs are represented by a ribbon, except for helix α2, which is drawn as a cartoon for visual reference. The OBDs are coloured from purple to red according to the distance to the central six-fold axis, where the OBD coloured purple is closest to that axis. (**A**) and (**B**) provide a lateral and a top view of the hexamer respectively, whereas (**C**) shows a view along the rotation axis relating the orientations of the OBDs.

We have previously described the overall structure of hexameric RepB (RepB_6_), showing that each protomer folds into two domains as displayed in Fig. 2 2. The C-terminal domain was found to hexamerise into a ring with six-fold symmetry and was subsequently called the oligomerisation domain (OD). Surprisingly, a symmetry mismatch occurs between the OD ring and the N-terminal origin-binding nuclease domain (OBD): the OD ring has C6, whereas the OBD ring has either a C2 or an approximate C3 symmetry depending on the crystal form (Fig. 1). The different overall symmetries in the structures occur as a consequence of a pronounced OBD displacement when comparing different protomers. In geometrical terms, this displacement can be described by a rotation around an axis at an angle of approximately 45° to the central C6 axis of the OD ring (Fig. 2C), which places the OBD at various distances from the central C6 axis. This leads to a total of nine distinct OBD orientations relative to the ODs in the two crystal structures solved, three for the C2 form and six for the C3 form (Fig. 2). The lack of a fixed orientation for the OBD can be explained by the poor or inexistent buried surface area (BSA) between consecutive OBD domains that varies greatly (Supplementary Table S1), indicating that the interactions are not cooperative and that the C6 symmetry is not favoured.

### Oligomeric state of the RepB OD- and OBD-only constructs

To confirm the distinct oligomerisation behaviour of the two RepB domains, both were expressed and purified separately, and subjected to sedimentation equilibrium and sedimentation velocity analytical ultracentrifugation (AU; Supplementary Fig. S1). At various concentrations of the OBD (10–60 μM), the sedimentation equilibrium gradient fitted well to an average molecular mass of 16,550 ± 500, which is compatible with the main species in the samples being the OBD monomer (sequence-derived molecular weight of 15,300 Da). No increase in the estimated molecular mass was observed as the protein concentration was augmented, indicating that the OBD does not self-associate in the analysed concentration range (not shown). The OBD-only construct sedimented as a main peak (>95% of the total absorbance) with a sedimentation coefficient (s) under standard conditions of 1.77 ± 0.1S, which is consistent with the monomeric form of the OBD. A tiny peak with a standard s-value of 4.5 ± 0.3S was also observed that could correspond either to OBD aggregates that were not in equilibrium with the monomeric form or to a trace contaminant. On the other hand, a single-species model accounts well for the sedimentation equilibrium gradient of OD, with an average molecular mass of 57,700 ± 200 that corresponds with the theoretical mass of the OD hexamer (58,300 Da). The OD sedimented as a single species with a standard s-value of 4.03 ± 0.1S and an estimated molar mass of 57,300. The protein concentration (in the 10–100 μM range) had no significant impact on the sedimentation velocity and equilibrium behaviour of the protein, which indicates that the OD hexamer has no tendency to form oligomers of higher molecular weight.

### Solution conformation of RepB_6_

**Figure 3:**
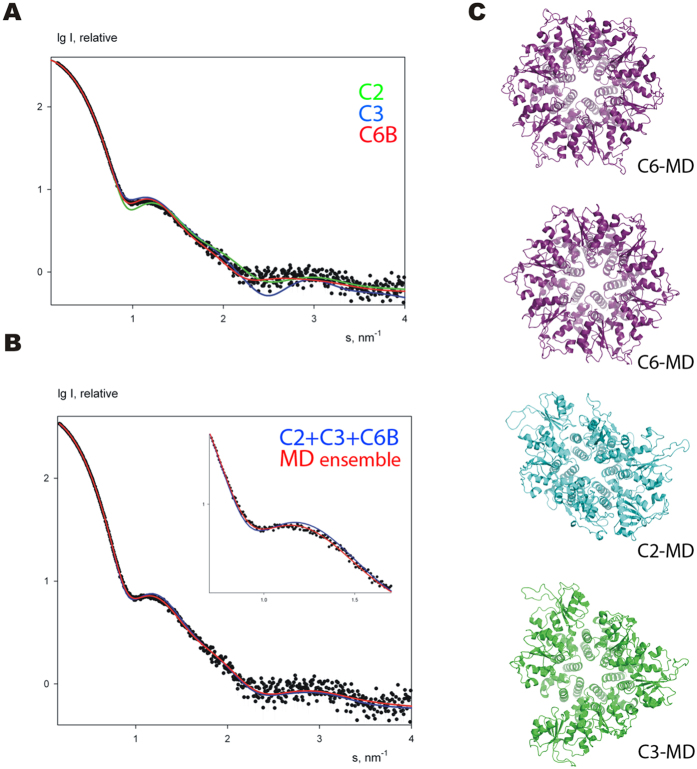
Experimental SAXS curve of free RepB in solution (black dots) and calculated fits (solid lines). (**A**) Fits from the individual atomic models C2, C3 and C6B are shown in green, blue and red, respectively. (**B**) Fits by the best mixtures of conformations: C2, C3 and C6B mix is shown in blue and the four representative MD snapshots (from panel **C**) in red. The inset emphasizes the differences in the fits in the region of the first shoulder of the SAXS profile. (**C**) Subset of four MD snapshots selected by EOM, which give the best fit according to OLIGOMER. The approximate symmetry of the models is indicated.

To compare the oligomerisation state and overall shape of RepB_6_ in the crystal structures and in solution, SAXS curves were recorded on the free protein at concentrations of 20, 40 and 83 μM of RepB_6_ (Fig. 3A). The scattering curves measured at different RepB concentrations were similar, showing that aggregation did not interfere in the analysis, and that the hexamer was not disrupted at concentrations down to 20 μM in solution, in accordance with the AU results. The maximum particle size was 105 Å, which fits well with the overall size of the C2 and C3 structures (Fig. 1A). The SAXS scattering curve with the best signal-to-noise ratio, obtained at 83 μM of RepB_6_, was used for further calculations.

The scattering profiles calculated by the program CRYSOL^17^ from atomic models of C2 and C3 did not provide good fits to the experimental data (Fig. 3A). There are systematic deviations in the region of the first shoulder, and the resulting discrepancy (χ) values are 2.2 for both structures. Representing the sample as a mixture of C2 and C3 states in program OLIGOMER^18^ allowed only marginal enhancement of the fit (χ = 1.8, Supplementary Fig. S2). In an attempt to improve the fits, nine models with overall C6 symmetry were generated by applying the C6 symmetry of the OD to each of the nine OBD conformations of the crystal structures. The C6 symmetrised model derived from chain B of the 3DKX (C2) structure resulted in a compact hexamer without severe interdomain clashes (C6B, Fig. 1A). For all other OBDs, hexamerisation led to unsatisfactory models containing either unlikely gaps or severe clashes between adjacent OBDs. In addition, only the C6B model gave a reasonable fit to the SAXS curve (χ = 1.6, Fig. 3A).

Interestingly, the C6B model gives a better fit than each of the crystal structures separately or any of their combinations, but its calculated scattering profile demonstrates a somewhat flat behaviour in the region of the secondary maxima (s ~3 nm^−1^; Fig. 3A) where the crystallographic C2 model is superior over the symmetrised one. A non-significant improvement was obtained for a ternary mixture of C2, C3 and C6B at 0.3:0.15:0.55 stoichiometry, which gave a χ of 1.5 (Fig. 3B). The flexibility implied by the poor BSA and the different orientations of the OBDs observed in the crystal structures suggests that the C6B fits well to the experimental data because its hexameric form represents an average structure of an ensemble of non-C6 conformations. To obtain more detailed structural information from the SAXS data and to improve the fit with them, we have employed computational techniques to generate an extensive ensemble of different OBD conformations for comparison with SAXS.

### RepB conformational plasticity

We used C_α_-based Normal Mode Analysis (NMA) to study the directionality of the movements of the OBDs, treating the C2 and C3 X-ray structures and the C6B model as stable reference states. The simulation models include residues E4-G204 of the RepB sequence. Initial visual inspection of the preferred displacements along the first five normal modes (trajectories can be found at http://mmb.pcb.ub.es/RepB) confirmed that the largest displacements were localized in the OBDs, while the OD ring remained largely unchanged. The dot (scalar) products between the first two normal modes of C2-C3, C2-C6B, and C3-C6B transitions were 0.55, 0.79, and 0.61, respectively, showing that the protein has the tendency to move in similar directions. These similarity values are surprisingly high, with associated Z-score values > 1500. Interestingly, C2-C3 overlap was lower than those of C2-C6B and C3-C6B, suggesting that the C6B displays intermediate directionality pattern between C2 and C3 states. This transitory nature of the C6B model can be understood, because it is derived by hexamerisation of the protomer (Fig. 1A) that has the OBD positioned roughly halfway in the trajectory shown in Fig. 2 (see above).

We then compared the similarity between the observed motions in the crystal structure (defined by the transition vector *T*, see Methods) and the principal eigenvectors obtained from the NMA of the different structures. This study showed how well the intrinsic dynamics of a protein is programmed to drive a conformational transition. Results on Table 1 indicated that transitions between C2 and C6B symmetries (involving a synchronic movement of 2 + 2 OBD protomers; 7.24 Å C_α_ RMSD difference) were implicitly coded in the structure of both conformers, and suggested that the OBDs tend to be displaced along the almost linear transition observed in the X-ray structures (see Fig. 2 and http://mmb.pcb.ub.es/RepB). Thus, the C2*C6B transition was mainly dictated by the first C2 normal mode, which explains ~60% of the motion, whereas explaining the same amount of variance for the C6B*C2 transition required two modes (~60% of the motion). Such overlaps were extremely significant compared with a random one (see Table 1). The transition C2*C3 (10.3 Å C_α_ RMSD) and C3*C6B (8.5 Å C_α_ RMSD) shared a common route, as shown by the scalar product between their respective transition vectors (*T*_C2*C3_ • *T*_C3*C6B_ = 0.51), whereas the C2*C3 and C2*C6B routes seemed more distinct (*T*_C2*C3_ • *T*_C2*C6B_ = 0.26), and both routes involving C6B seem to be quite different (*T*_C2*C6B_ • *T*_C3*C6B_ = 0.05). Taken all the results together, we can summarize that the transition C2*C6B seems the most likely to happen, followed by the transition C2*C3, being the transition C3*C6B (involving large movement of 4 OBD protomers) the least likely to occur.

### Generation of the MD ensembles

**Figure 4:**
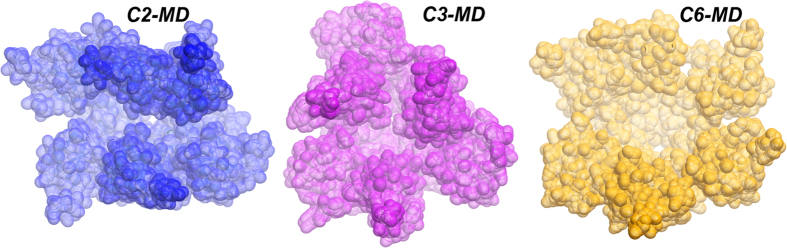
Average structures obtained from the last 10 nanoseconds of 100 nanoseconds long MD started from the C2 (3DKX), C3 (3DKY) and C6B (model) structures.

To study in further detail the movement and transient associations between the OBDs in solution we performed three long (100 nanoseconds) atomistic molecular dynamics (MD) in explicit solvent, one for each conformation (C2, C3, C6B model). In all three simulations, the OBDs moved considerably during the trajectories as reflected by their final RMSD with respect to their starting X-ray structures (C2 = 8.41 Å, C3 = 8.51 Å, C6B = 9.56 Å) (see Supplementary Fig. S3; note that we included rotational symmetry in the RMSD calculation^19^). These values are clearly higher than those expected for a globular protein of this size^20^,^21^, suggesting high flexibility for these systems. Despite such a high mobility, we could not observe full conversions among the C2, C3 and C6B structures (cross-RMSD values were ~10 Å) in 100 nanoseconds MD. Yet this observation suggests lack of preferred cooperative interactions between the OBDs, it may be possible that these transitions occur in much longer time scales. Interestingly, despite the idealized C6B form was that providing the best individual fit to the SAXS curve, none of three trajectories converged to a form with C6 symmetry between protomers, not even the one starting from the C6B model (see average structures in Fig. 4 and trajectory movies at http://mmb.pcb.ub.es/RepB). On the other hand, visual inspection of the trajectories showed that OBDs displayed tendency to form dimers and we found that transitions occurred at the dimer-level, with the exception of C3*C6B (see RMSD values at the Supplementary Fig. S4).

### Comparison of MD ensemble and SAXS data

We used snapshots from the three MD trajectories to check whether we could improve the fit to the experimental SAXS curve. For this purpose, we used the Ensemble Optimization Method (EOM), an approach that uses a genetic algorithm to select representatives from a large pool of structures that best fit to the experimental SAXS curve of a flexible protein^22^. In our case, the EOM heuristic search selected 12 models (from 3000 MD snapshots), which resulted in a χ value of 1.2 using the full data range (0–0.5 s). Post-processing of the selected ensemble with OLIGOMER showed that a yet smaller subset is sufficient to fit the experimental data. A mixture of four structures (Fig. 3C) yields the fit (red line in Fig. 3B) with the same χ value as that of EOM, whereby the volume fractions of C6B-, C3- and C2-like models are, respectively, 0.55, 0.35 and 0.10. These results agree with the comparison of the three rigid models to the SAXS data (see above) and further confirm conformational variability of RepB in solution.

### Structural elements controlling OBD relative orientation

**Figure 5:**
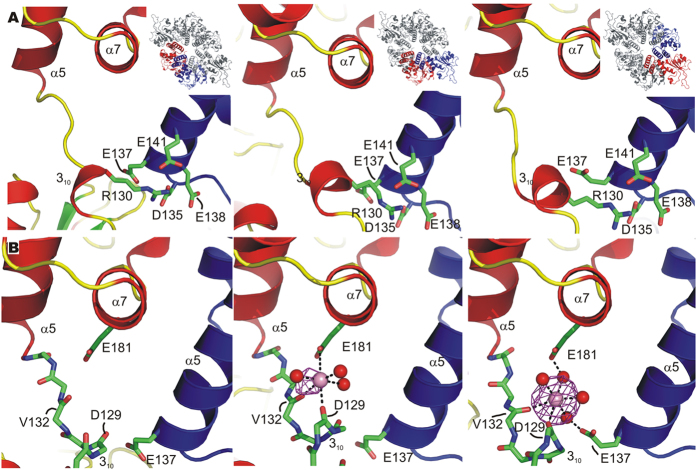
The control elements governing OBD movement in the RepB X-ray structures. (**A**) The location of R130 at the OD/OBD interface and of the D and E residues of the N-terminal end of helix α5 that interact with R130 is shown. The insets show the position of the protomers in the C2 hexamer. (**B**) Views on the region where OBD conformation-dependent metal binding occurs at the different OD/OBDs interfaces of the structure cocrystallised with BaCl_2_. The protein chain is represented by a cartoon drawing in all panels, except for the interdomain loop region (residues 128–134) containing the 3_10_ helix, for which only a stick representation of the protein backbone is shown. The non-interacting side chains are left out for clarity. The neighbouring protomer is coloured blue and the Ba^2+^ ions are represented by a pink sphere. The contours represent the Ba^2+^ anomalous map (see text) contoured at 5σ. Direct and indirect contacts between protein residues and the metal ion, when present, are indicated by dashed lines. The OBD and OD are from two adjacent protomers and are colour-coded as in panels (**A**).

The position and freedom of movement of the OBDs is mainly determined by the OBD-OD hinge region, since contacts between adjacent OBDs do not seem to play an important role in fixing their positions (see above). We therefore analysed this hinge region in detail and observed two features important for OBD fixation. Due to the low resolution of the C3 form, this analysis is performed for the C2 form only. First of all, a salt bridge is formed between R130 of the 3_10_ helix of the hinge region and a patch of four negatively charged residues (D135, E137, E138 and E141) of the OD helix α5 of the neighbouring protomer (Fig. 5A). The salt bridge is possible because the counterclockwise rotation of the OBDs relative to the OD ring (Fig. 1B) allows the residues involved to be positioned correctly for interaction. The size of the patch of negatively charged carboxylate moieties allows the neighbouring N-terminal domain to change position without disruption of the salt bridge. Additional salt bridges between OBD and OD residues of adjacent protomers, *i.e.* R7 with E137 and K63 with D135, form in certain orientations where the OBD is turned away from the central hexameric axis of the ODs (blue coloured OBDs of the C3 form in Fig. 1B).

A structural element that may contribute to stabilize the outward position of the OBD is the presence of a metal ion bound in this area. In the previously published C2 structure (3DKX)^2^ strong residual electron density was observed in the area and assigned to a Mg^2+^ ion with octahedral coordination in one of the three protomers of the crystal asymmetric unit, the one with the ODB in an outward position. The coordination of the metal ion is provided by backbone carbonyl oxygen atoms of the 3_10_ helix of the hinge region and the side chain of residue E181 of helix α7 of the same protomer. In addition, E137 (OD helix α5) of an adjacent protomer interacts with the metal ion through a bridging water molecule instead of making a salt bridge with R7, as when the OBD is closer to the C6 axis (inward and intermediate positions). To confirm the metal ion binding, we co-crystallised RepB_6_ with Ba^2+^ and exploited the X-ray absorption of the Ba^2+^ ion. The anomalous difference map shows that the Ba^2+^ occupies the same position assigned previously to a Mg^2+^ ion. Some density was also observed in the protomer where the OBD is in an intermediate position, and was refined as a Ba^2+^ ion with half occupancy (Fig. 5B). However, the presence of divalent metal ions in the medium does not seem to be determinant in driving the hexamer towards the C2 conformation, as Mg^2+^ was also used in the crystallization solution of C3 (even though no divalent cation could be located in this structure). AU experiments have not revealed either any difference in the oligomerization state of RepB_6_ when metal ions are present or absent (not shown).

### A potential OD is universally present in the hexameric viral Rep proteins

**Figure 6:**
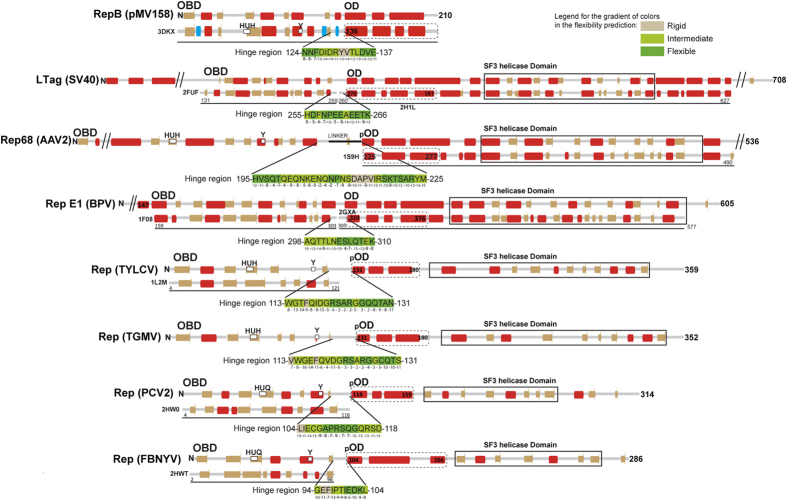
Predicted and observed secondary structure of the potential or defined domains of the pMV158 and viral Rep proteins discussed in the main text. The amino-terminal end (N) and the number of amino acids are indicated for each protein analysed. The top horizontal line shows the result of the secondary structure prediction (predicted α-helices and β-strands are respectively represented by red and brown bars). The bottom horizontal line shows the observed secondary structure for the protein fragments whose structure is available (α-helices, β-strands and 3_10_-helices are respectively represented by red, brown and blue bars). Conserved amino acid residues of the active site involved in metal binding (HUH) and in the endonucleolytic activity are indicated in the protein maps. The limits of the OBD, OD (either confirmed (OD) or potential (pOD) domain) and of the helicase domain are indicated in the protein maps. For RepB, AAV2 Rep, BPV E1 and SV40 LTag, the observed secondary structure of the ODs from the crystal structures are shown below the predicted secondary structure, preceded by the PDB entry code. Since there are no OBD structures available for the AAV2 and TGMV Reps, the C-terminal boundary of the OBD is shown as defined by alignment with the PF08724 and PF00799 Pfam families, respectively. Helicase domains of FBNYV and PCV2 Reps are defined by alignment with Pfam family PF00910 of SF3 helicases. The sequence and coordinates of the OBD-OD interdomain region that constitutes a potential hinge is indicated for every protein. The flexibility of each residue of the hinge predicted by the PredyFlexy^35^ server is represented by the gradient of colours indicated in the upper right corner of the figure. Grey colour represents a rigid residue whereas green and dark green colours represent residues with intermediate or high flexibility, respectively. The confidence index for the flexibility prediction is indicated for each residue just below the sequence. This index reflects the accuracy of the prediction and is represented by discrete values ranked from 1 to 19, with the prediction confidence increasing.

**Figure 7:**
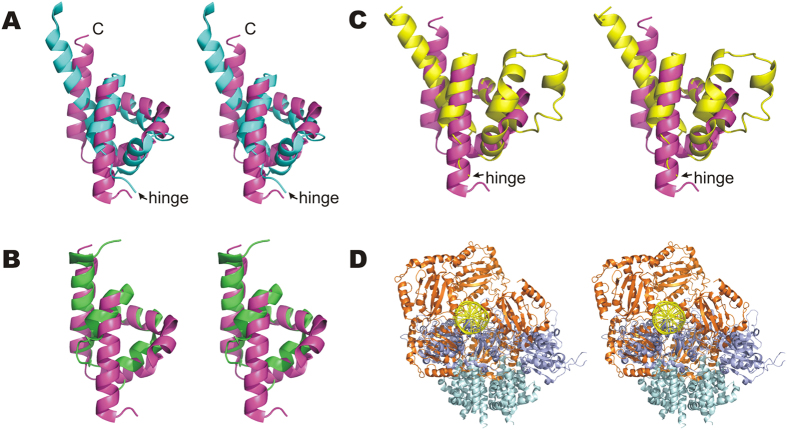
Comparison between the RepB structure and the structures of the viral initiators. (**A**–**C**) Stereo views of the superposition of the ODs of BPV E1 helicase (PDB entry 2GXA, residues 308–378, light blue), AAV2 Rep (1S9H, residues 223–280, green) and SV40 LTag (1SVM, residues 266–355, yellow) on the RepB C2 structure (3DKX, residues 132–203, magenta). Superpositions were calculated using the SSM algorithm^62^. For BPV E1 and SV40 LTag, the OBD-OD random-coiled hinge region is indicated. (**D**) Stereo view of a cartoon representation of the superposition of chain B of the C2 RepB hexamer on one of the OBD monomers of the AAV5 Rep in complex with its origin. AAV5 OBDs are coloured orange, the AAV5 DNA green, the RepB OBDs dark blue and the RepB ODs light blue.

Given the resemblance of pMV158 RepB with Rep initiators of viral origin, it was interesting to compare the domain composition and interdomain flexibility of these proteins. The PDB currently holds structures of protein fragments comprising the OBD or the helicase domain of Reps of the ssDNA geminiviruses tomato yellow leaf curl virus (TYLCV) and tomato golden mosaic virus (TGMV), the ssDNA nanovirus faba bean necrotic yellows virus (FBNYV), the ssDNA porcine circovirus type 2 (PCV2), the ssDNA parvoviruses human bocavirus (HBV) and adeno-associated virus 2 and 5 (AAV2 and AAV5), the dsDNA polyomavirus simian virus 40 (SV40) and the dsDNA bovine and human papillomaviruses (BPV and HPV.) An all-helical region comprising 3 to 5 α-helices is predicted to exist between the OBD and the helicase domain of each of the above Rep proteins (Fig. 6). Structurally, it constitutes a separate domain in Reps for which the atomic structure of this region is available^12^,^23^,^24^. These intermediate regions of the viral proteins resemble the RepB OD in both their α-helical composition and their location C-terminal to the OBDs, although their role in hexamerisation has only been unambiguously defined for the initiators E1 of BPV^12^ and large tumour antigen (LTag) of SV40^24^,^25^. Separate OBDs of viral Rep proteins are generally purified as monomers, whereas the full length proteins and N-terminal truncations do form hexamers^13^. The structural models also reflect the monomeric state of the OBD domains (PDB codes: *AAV5*: 1M55, 1RZ9, 1UUT; *TYLCV*: 1L5I, 1L2M; *FBNYV*: 2HWT; *PCV2*: 2HW0; *HBV*: 4KW3; *E1 helicase*: 1KSX, 1KSY, 1F08; *SV40 LTag*: 2NTC, 2IPR, 2ITJ, 2ITL, 2NL8, 1TBD). The only exception is a particular structure of the OBD of SV40 LTag (PDB code 2FUF) where the subunits arrange into a helical hexamer resembling a split lockwasher, which also lacks proper C6 symmetry. Remarkably, the viral helicase domains are also prone to form monomers when the interactions between the confirmed or potential ODs are disrupted either by point mutations or deletions (SV40 LTag^25^, E1^12^,^26^, AAV2^23^,^27^,^28^, TYLCV^13^, TGMV^29^). Interestingly, the interdomain linker preceding the potential OD’s helical bundle of AAV2 and AAV5 Rep protein is required for hexamerisation^30^,^31^, suggesting that the N-terminal region of the potential OD is most important for its function, an observation that is consistent with the contacts between the RepB hinge region and neighbouring ODs (see above). Since the ODs of RepB and the viral Reps share a similar function and a similar fold (Fig. 7A–C), they most likely share a common ancestor. It should be noted, however, that the orientation in the hexameric ring has changed considerably in the different Reps.

For the ssDNA nanovirus and circovirus Reps, only the OBD structure is available (Fig. 6) and an OD domain has not yet been confirmed experimentally for these proteins^32^. However, a sequence-based secondary structure prediction performed in this work using Jpred^33^ suggests that such a domain may exist for these Rep proteins (Fig. 6). The C-terminal helicase domains of these initiators are annotated to belong to the Pfam^34^ family of 2C helicases (entry PF00910), in contrast to the helicase domains of the other viral helicases discussed here. The 2C-like helicase domains of the ssDNA nano- and circoviruses were suggested to originate from ssRNA caliciviruses on the basis of homology with part of the calicivirus 2C helicases^35^. Interestingly, secondary structure predictions with Jpred^33^ of the 2C helicases from several hexameric ssRNA picornaviruses (e.g. bovine foot-and-mouth-disease, human parechovirus 30, human coxsackie virus and the human poliovirus), containing a 2C helicase domain but lacking the OBD, show a N-terminal ~90 residue stretch rich in α-helices immediately following a N-terminal helix required for association of the 2C domain with membranes. These parts of the 2C helicases are not considered to be part of the PF00910 SF3 helicase domain and our preliminary results therefore indicate that the presence of OD domains may be a common feature in hexameric replication-associated viral helicases of the SF3 family and call for a more exhaustive search for ODs among the SF3 family members.

### A potential flexible hinge region connecting OBD and OD is universally found in hexameric Rep proteins

Given the importance of the hinge region for OBD movement and perhaps for hexamerisation, we addressed the question whether the OBD-hinge-OD arrangement is also present in the rest of the initiators of the pMV158 family and in viral Reps. Sequence alignment of Reps within the pMV158 family shows that this is the case for these related proteins (Supplementary Figs S5 and S6). To compare viral Reps and RepB only predictions on secondary structure and local flexibility were used^36^,^37^, because full sequence alignments are hampered by the low sequence similarity between the Reps (Fig. 6). This analysis revealed a 10–31 residue unstructured region between OBD and OD in all viral helicases discussed herein (Fig. 6). Moreover, a number of residues within these unstructured regions are predicted to be highly flexible with a good confidence index using the PredyFlexy server^36^. The presence of a hinge is supported by the available experimental structures of viral Reps containing the OD, which consistently show the presence of an unstructured amino acid stretch preceding the OD, some examples of which are shown in Fig. 7. Furthermore, the structure of a SV40 LTag double hexamer loaded onto the viral replication origin determined by electron microscopy suggested that a hinge region consisting in a 12-residue flexible linker allows mobility of the OBDs relative to the helicase domains (which include the OD subdomains) in each hexamer^38^. Given the preservation of the OBD-hinge-OD architecture within the hexameric Reps (Fig. 6), it seems likely that this arrangement has been exchanged as a single unit between replicators throughout evolution. However, the presence of the OD in the OBD-lacking 2C helicases (see above) indicates that the OD has also been exchanged in absence of the OBD.

### Importance of the hinge region for DNA interactions of the initiators

RepB is able to bind with high affinity to a set of three tandem 11-bp direct repeats and to nick the plasmid DNA at a site located ~80 bp apart, within an inverted repeat hairpin loop^1^,^39^. RepB also binds with lower affinity to a set of two tandem 7-bp direct repeats that are 3′ adjacent to the inverted repeat of the replication origin^39^. Interaction of the plasmid initiator with these three different sequences might play a crucial role in activating the replication origin. Similarly, AAV5 Rep can also recognize different DNA regions of distinct topology, *i.e.* a five 4-bp direct repeat sequence, a hairpin loop containing the nick site, and one of the terminal hairpin arms at the inverted terminal repeats that constitute the viral replication origin^15^. The structure of the AAV5 OBD in complex with the direct repeat sequence has been reported (Fig. 7D)^15^. The arrangement of OBDs in this structure is not compatible with the hexameric topology of the OD. The structure may represent an intermediate in the assembly of a hexameric initiation complex^15^. Such an assembly process in which the formation of the hexamer is induced by DNA binding has been proposed for a number of viral Rep proteins of the SF3 superfamily^11^,^27^,^40^. Alternatively, it is possible that the DNA changes its conformation upon sequential binding of the protomers of a pre-formed hexamer of the initiator. In any case, the complex formation between the hexameric initiator and dsDNA is only possible if the DNA is significantly distorted in the process. Consistently, RepB binding to the *dso* DNA of the pMV158 plasmid significantly bends this DNA^39^. In both assembly pathways, the hinge region would play a pivotal role.

Overall, the results presented here confirm the RepB-OBD flexibility in solution. Its structure in solution is represented by the ensemble of four structures shown in Fig. 3C. We show that OD rings similar to that of RepB could be present in viral Reps, where they would allow the helicase domains to stably encircle the DNA and processively unwind the DNA by ATPase-dependent rearrangements of the helicase domains^41^. The OD thus provides a rigid scaffold for the N- and C-terminal domains that need a certain level of conformational flexibility important for function^42^. In addition, we show that an OBD-OD hinge region may exist in viral Reps, which would confer the ability to process the DNA appropriately by facilitating the recognition of a variety of different DNA structures and topologies^15^,^43^. Moreover, severe distortion of the DNA might facilitate simultaneous binding of a Rep protein to all distant recognition sites in the origin DNA. In order to fully understand the atomic mechanism of replication initiation in these systems, an important future challenge is to determine the structure of the protein-DNA complex required for the onset of replication.

## Methods

### X-ray diffraction

Crystals of the C2 form were grown by sitting drop vapor diffusion against a crystallization buffer containing 50 mM TRIS pH 8.5, 100 mM BaCl_2_, 12% PEG 8K. Crystals were transferred to crystallization buffer supplemented with 20% glycerol and flash-frozen in liquid nitrogen. Data were collected to 3.8 Å on BM16 (ESRF) near the L-edge of Ba (E = 5.9890 keV) and reduced using the XDS package^44^ (see Supplementary Table S2). A restrained refinement calculation was performed starting from the C2 structure (PDB code 3DKX) free of its water molecules, applying NCS restraints on the OBD (up to residue D129) and OD (residues D135-C terminus) domains and including TLS parameters for the N-terminal residues up to L134 and for the three OD domains (residues 135-C terminal), using the Phenix.refine program v1.6^45^. A single B factor was refined for each residue in the structure. After each refinement step, the fit of the model with the density map was visualized with Coot^46^ and the geometry was checked using Molprobity^47^. The anomalous map was calculated using the FFT program from CCP4 package^48^, using the anomalous differences and the phases from the final refinement as coefficients. Barium atoms were identified based on strong densities observed in the 2F_o_-F_c_ and F_o_-F_c_ maps and in anomalous difference maps. The final model comprised residues 3–204 for the three protomers, except for chain A, for which density residues K43-K52 were removed. Final *R*_*cryst*_ and *R*_*free*_ are 20.5% and 23.7%, respectively. The final model was validated using Molprobity^47^ (see Supplementary Table S2). The structure and structure factors were deposited in the PDB with entry code 4U87.

### Generation of C6 symmetrised models

The C-terminal domains of each of the respective nine protomers of the two crystal structures with PDB codes 3DKX and 3DKY were superimposed on the six domains of the invariant six-fold C-terminal ring. This procedure results in nine models with six-fold symmetry for the full-length protein, where in each respective model the N-terminal domains have the same orientation with respect to the C-terminal domain.

### Small Angle X-ray Scattering (SAXS)

RepB was prepared as described previously^3^ and brought to a concentration of 12.1 mg/mL in a buffer of 10 mM TRIS pH 8.5, 5 mM EDTA, 1.0 M KCl. Data were measured at the EMBL X33 beamline at the DESY synchrotron (Hamburg, Germany) covering a momentum transfer range of 0.06 < s < 5.1 nm^−1^ (*s = 4πsin(θ)/λ*, where *2* *θ* is the scattering angle and *λ* is the X-ray wavelength). Two frames of 60 s each were collected and averaged after ensuring that the sample did not suffer from radiation damage. Similar data were collected on samples diluted to 5.8 and 2.9 mg/mL. The data were processed using the PRIMUS program of the ATSAS package^18^ giving a radius of gyration (*R*_*g*_) of 38 ± 0.5 Å. The distance distribution function, p(r), was calculated from the scattering patterns with GNOM^49^, from which the maximum particle dimension, D_max_, was determined as 105 Å. The program CRYSOL^17^ was used to produce calculated scattering data of both X-ray structures and models with C6 symmetry generated from the different protomers of the two X-ray structures. The stoichiometry that best fit the experimental data was calculated with OLIGOMER^18^ for various combinations of the X-ray structures, the derived hexamerised models and selected MD snapshots. The EOM approach^22^ has been applied to select representative conformations from MD trajectories that fit the experimental SAXS data.

### Normal mode analysis

NMA was performed according to the anisotropic network model approach (ANM)^50^ with *in-house* tools^51^. In the ANM, an Elastic Network Model^52^ is built for the C_α_ atoms, on which the force constant matrix (Hessian) is obtained from the partial second derivative of the potential with respect to the coordinates. The diagonalisation of the Hessian yields a set of eigenvectors ranked according to their associated eigenvalues. The first eigenvectors are usually related to the observed (experimental) biological motions, and they can be quantitatively compared to the transition between two states. The observed motion was numerically defined as the transition vector *T* (normalised) that is obtained by subtracting the coordinates of the final state to the initial one, after 3D superimposition. The correlation between the observed motion and the intrinsic deformations of RepB was estimated as the sum of the dot product of the first 2, 5, and 10 eigenvectors with *T*, as,


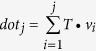


where *v* stands for the eigenvector, *i* for the eigenvector index, and *j* is the number of eigenvectors used (i.e., 5 for dot5).

### Molecular dynamics simulations

C2, C3 and C6B structures were processed (protonated, solvated, ionized, minimized and equilibrated for 200 picoseconds) using our standard MoDEL database procedure^21^. The simulations were extended to 100 nanoseconds using CHARMM22 force field^53^ at room temperature (T = 300 K) in the isothermal/isobaric ensemble, using periodic boundary conditions and particle Mesh Ewald corrections for the representation of long range electrostatic effects. All trajectories were generated using NAMD2 program^54^ at the *MareNostrum* supercomputer at the Barcelona Supercomputing Centre. Analysis of trajectories was performed using *ptraj*^55^, VMD^56^, ICM^57^, BioSuper web server^19^, as well as *in-house* software developed specifically for this project.

### Sedimentation equilibrium and sedimentation velocity

Sedimentation equilibrium experiments were performed at 20 °C in an Optima XLA (Beckman-Coulter) analytical ultracentrifuge equipped with UV–Visible absorbance optics, using an An50Ti rotor with standard 12-mm double sector or six-channel centrepieces of charcoal-filled Epon. RepB OBD (ranging in concentration from 10 to 60 μM) and RepB OD (ranging in concentration from 10 to 100 μM) in 20 mM NaH_2_PO_4_ pH 7.0, 150 mM NaCl, were centrifuged at sedimentation equilibrium at 22,000 and 13,000 rpm respectively. The equilibrium scans were taken at the most appropriate wavelength (230, 280 or 290 nm), depending upon the protein concentration. In all cases, the baseline signals were measured after high-speed centrifugation. Whole cell average molar masses were determined by fitting a sedimentation equilibrium model for a single sedimenting solute to individual datasets with the HeteroAnalysis software. The partial specific volumes of RepB OBD and OD were 0.755 and 0.737 ml/g respectively, calculated from the amino acid composition of the separate domains with the program SEDNTERP^58^.

Sedimentation velocity experiments were performed at 48,000 rpm and 20 °C in the same XLA analytical ultracentrifuge. Samples of RepB OBD (ranging from 10 to 60 μM) and OD (ranging from 10 to 100 μM) in 20 mM NaH_2_PO_4_ pH 7.0, 150 mM NaCl, were loaded into double sector centrepieces. Sedimentation profiles were registered every 5 min at the appropriate wavelength. The apparent sedimentation coefficient of RepB OBD and OD were calculated using the SEDFIT program^59^. This program generated apparent sedimentation coefficient distributions, c(S), by least squares boundary modelling of the sedimentation velocity data. The coefficients were corrected to standard conditions to get the corresponding S_20,w_ values using the SEDNTERP program. From the combined data for the OBD, a frictional ratio of 1.33 ± 0.1 was calculated, which indicates that the hydrodynamic behaviour of the OBD monomer somewhat deviates from the one corresponding to a rigid spherical particle. The frictional ratio calculated for the OD hexamer is 1.33 ± 0.1, also indicating differences in the hydrodynamic behaviour respect to a rigid spherical particle of the same molar mass.

### Purification of the RepB OBD and OD domains and removal of N-terminal His-tags

The separate RepB domains were purified as described previously^2^. The N-terminal 6xHis-tagged RepB OBD (residues 1 to 132) and RepB OD (residues 127 to 210) were overproduced in *Escherichia coli* M15 and purified by metal-ion affinity chromatography using Ni-NTA agarose (His-Select SIGMA). The N-terminal His-tags of OBD and OD were completely removed by using the exoproteolytic enzymes of the TAGZyme system (Unizyme).

### Sequence alignments and secondary structure and flexibility predictions

Sequences of viral initiators were retrieved from the UniprotKB server (http://www.uniprot.org/) and visualized with JALVIEW^37^. Secondary structure predictions were performed using the Jnet application^33^. The boundaries of the OBD, OD and helicase domains in the primary sequence were identified based on the atomic structures, or by alignment with the domain families defined in the Pfam database when the structure was not available. Flexibility prediction of the sequence encompassing the OBD-OD hinge region was performed with the PredyFlexy server^36^.

### Miscellaneous

Structure comparisons and superpositions were performed using the molecular graphics program Coot^46^. Figures were prepared using PyMOL (The PyMOL Molecular Graphics System, Version 0.99, Schrödinger, LLC) unless otherwise stated. The BSA between adjacent protomers was calculated using PISA^60^. The 3D morphing transitions between conformations were created with Molsoft LLC’s ICM-Pro 3.7^57^.

SAXS data and selected MD models are deposited in the SASBDB database, accession code SASDBC3 (http://www.sasbdb.org/data/SASDBC3/).

Supplemental movies for the MD trajectories, morphing transitions between C2, C3 and C6, as well as Normal Mode projections and other 3D representations can be found at our website at http://mmb.pcb.ub.es/RepB. The 3D interactive objects can be visualized online by using the activeICM/active X plugin^61^ or downloaded as a single file to be browsed with all its attached objects locally with the ICM browser. Both activeICM and ICM browser are freely available to the public.

## Additional Information

**How to cite this article**: Boer, D. R. *et al.* Conformational plasticity of RepB, the replication initiator protein of promiscuous streptococcal plasmid pMV158. *Sci. Rep.* **6**, 20915; doi: 10.1038/srep20915 (2016).

## Supplementary Material

Supplementary Information

## Figures and Tables

**Table 1:** Dot product between the observed motions and the Normal Modes of C2 (3DKX), C3 (3DKY), and C6B (model) forms of RepB.

	*C2	*C3	*C6B
C2	—	0.19/0.49/0.51	0.59/0.60/0.72
C3	0.23/0.52/0.63	—	0.19/0.52/0.61
C6B	0.63/0.64/0.64	0.02/0.35/0.39	—

The numbers correspond to the dot product obtained when the essential space is defined by 2, 5 or 10 eigenvectors (dot2/dot5/dot10) as explained in the Methods section. Dot products expected by chance are 0.00055, 0.0014 and 0.0028 for 2, 5 and 10 eigenvectors respectively.
