# Supplementary material for: Conformational plasticity of RepB, the replication initiator protein of promiscuous streptococcal plasmid pMV158
Source: Sci Rep. 2016 Feb 15;6:20915. doi: 10.1038/srep20915 (PMC4753449; doi:10.1038/srep20915)
Supplement: Supplementary Information [file srep20915-s1.doc]

**Supplementary material for**

**Conformational plasticity of RepB, the replication initiator protein of promiscuous streptococcal plasmid pMV158**

D. Roeland Boer^1,2,$^, José Angel Ruiz-Masó^3,+^, Manuel Rueda^1,+^, Maxim V. Petoukhov^4^, Cristina Machón^1,2^, Dmitri I. Svergun^4^, Modesto Orozco^1,5^, Gloria del Solar^3,*^, Miquel Coll^1,2,*^

^1^ Institute for Research in Biomedicine (IRB Barcelona), Barcelona, 08028 Spain.

^2^ Institut de Biologia Molecular de Barcelona (Consejo Superior de Investigaciones Científicas), Barcelona, 08028, Spain.

^3^ Centro de Investigaciones Biológicas (Consejo Superior de Investigaciones Científicas), Madrid, 28040, Spain.

^4^ European Molecular Biology Laboratory, Hamburg Unit, EMBL c/o DESY, Hamburg, 22607, Germany.

^5^ Departament de Bioquímica, Facultat de Biologia, Universitat de Barcelona, Barcelona, 08028, Spain.

^$^ Current address: CELLS-ALBA Synchrotron Light Source, Carretera BP 1413 km3.3, Cerdanyola del Vallès, Spain.

^+^ Contributed equally

* Corresponding authors: MC: Institute for Research in Biomedicine and Institut de Biologia Molecular de Barcelona (CSIC), Barcelona Science Park, Baldiri Reixac 10-12, 08028 Barcelona, Spain. E-mail: miquel.coll@irbbarcelona.org. Phone: +34 93 4034951. Fax: +34 93 4034979. GS: Centro de Investigaciones Biológicas (CSIC), Ramiro de Maeztu 9, 28040 Madrid. Spain. E-mail: [gdelsolar@cib.csic.es](mailto:gdelsolar@cib.csic.es). Phone: +31 91 8373112

**Supplementary Table S1.** Summary of the buried surface areas between adjacent protomers of the C2 and C3 RepB structures, differentiating the OBD and OD interfaces.

|  | C2 OD Interface area (Å) | C2 OBD Interface area (Å) | C3 OD Interface area (Å) | C3 OBD Interface area (Å) |
| --- | --- | --- | --- | --- |
| A-B | 696.1 | 310.4 | 747.6 | 414.2 |
| B-C | 695.1 | 404.4 | 733.4 | 210.9 |
| C-D | 693.3 | 6.1 | 741.5 | 444.0 |
| D-E | 696.2 | 311.0 | 726.5 | 0 |
| E-F | 696.7 | 404.4 | 733.2 | 402.2 |
| F-A | 691.1 | 6.3 | 726.5 | 43.0 |
| SUM | 4168.5 | 1442.6 | 4408.7 | 1514.3 |

**Supplementary Table S2.** Summary of the data processing and refinement statistics of the C2 crystal form grown in the presence of BaCl_2_.

| λ (Å ) | 2.0702 | R_cryst_^d^ /R_free_^e^ (%) | 20.5/23.7 |
| --- | --- | --- | --- |
| Space group | *P*3_2_21 | r.m.s. deviation from target values: |  |
| Unit cell parameters (Å) | *a*=*b*=85.8, *c*=246.2 | Bond lengths (Å) | 0.010 |
| Resolution range (Å) ^a^ | 19.77-3.8 (4.0-3.8) | Bond angle distances (Å) | 1.17 |
| # of reflections: |  | Molprobity scores: |  |
| total | 114166 (16256) | Clashscore (%ile) | 25.1 (89) |
| unique | 19844 (2858) | Poor rotamers (%) | 0 |
| Completeness (%) | 99.3 (100%) | Ramachandran Outliers (%) | 0 |
| <I / σ(I)> | 7.54 (2.82) | Ramachandran Favoured (%) | 94.9 |
| Average multiplicity | 5.75 (6.9) | Overall score (%ile) | 2.24 (100) |
| R_sym_ (%) ^b^ | 23.6 (73.1) | Average model B-factors (Å^2^) | 109.1 |
| R_meas_ (%) ^b^ | 26.0 (80.5) | B-factor from Wilson plot (Å^2^) | 67.2 |

^a^ Throughout the table, the values in parentheses are for the outermost resolution shell.

^b^ R_sym_ = Σ_h_ | Î_h_ – I_h,i_ | / Σ_h_Σ_i_ I_h,i_ , where Î_h_ = (1/n_h_) Σ_i_ I_h,i_ and n_h_ is the number of times a reflection is measured.

^c^ R_meas_ = [Σ_h_ (n_h_/[n_h_-1])^½^ Σ_i_ | Î_h_ – I_h,i_ |] / Σ_h_Σ_i_ I_h,i_ , where Î_h_ = (1/n_h_) Σ_i_ I_h,i_ and n_h_ is the number of times a reflection is measured.

^d^ R_cryst_ = Σ_hkl_ | |F_obs_| - k |F_calc_| | / Σ_hkl_ |F_obs_|

^e^ R_free_ = Σ_hkl⊂T_ | |F_obs_| - k |F_calc_| | / Σ_hkl⊂T_ |F_obs_| where T represents a test set comprising ~5% of all reflections excluded during refinement.

^f^ Calculated using the CCP4 program SFCHECK.


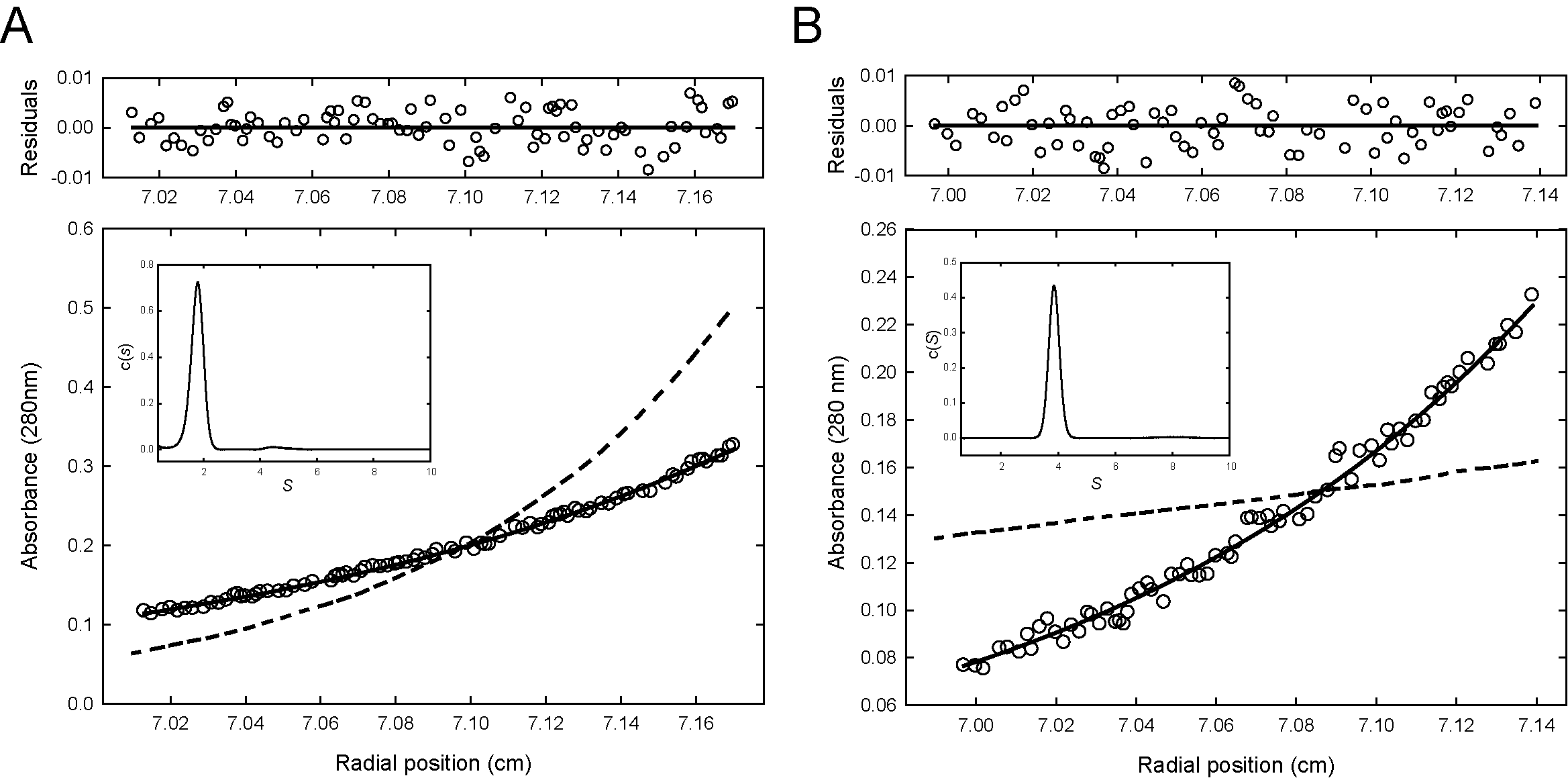


**Supplementary Figure S1.** Analytical ultracentrifugation profiles of the separate RepB domains. (**A**) sedimentation equilibrium gradient of RepB OBD (10 µM protein in 20 mM NaH_2_PO_4_ pH 7.0, 150 mM NaCl) at 22,000 rpm and 20ºC. Closed circles represent the experimental data, and the solid line is the best fit gradient, with an average molar mass of 16,800±100, which corresponds with the OBD monomer mass; the dashed line represents the theoretical gradient of an OBD dimer (30,600 Da). *Inset*, apparent sedimentation coefficient distribution, c(S), at 48,000 rpm and 20ºC, for the same sample of RepB OBD shown in the main figure. (**B**) sedimentation equilibrium gradient of RepB OD (20 µM protein in 20 mM NaH_2_PO_4_ pH 7.0, 150 mM NaCl) at 13,000 rpm and 20ºC. Closed circles represent the experimental data, and the solid line shows the best fit gradient to a single sedimenting species with an average molar mass of 57,700±200, which agrees with the OD hexamer mass; the dashed line is the theoretical gradient of an OD monomer (9,730 Da). *Inset*, apparent sedimentation coefficient distribution, c(S), at 48,000 rpm and 20ºC, for the same sample of RepB OD shown in the main figure.


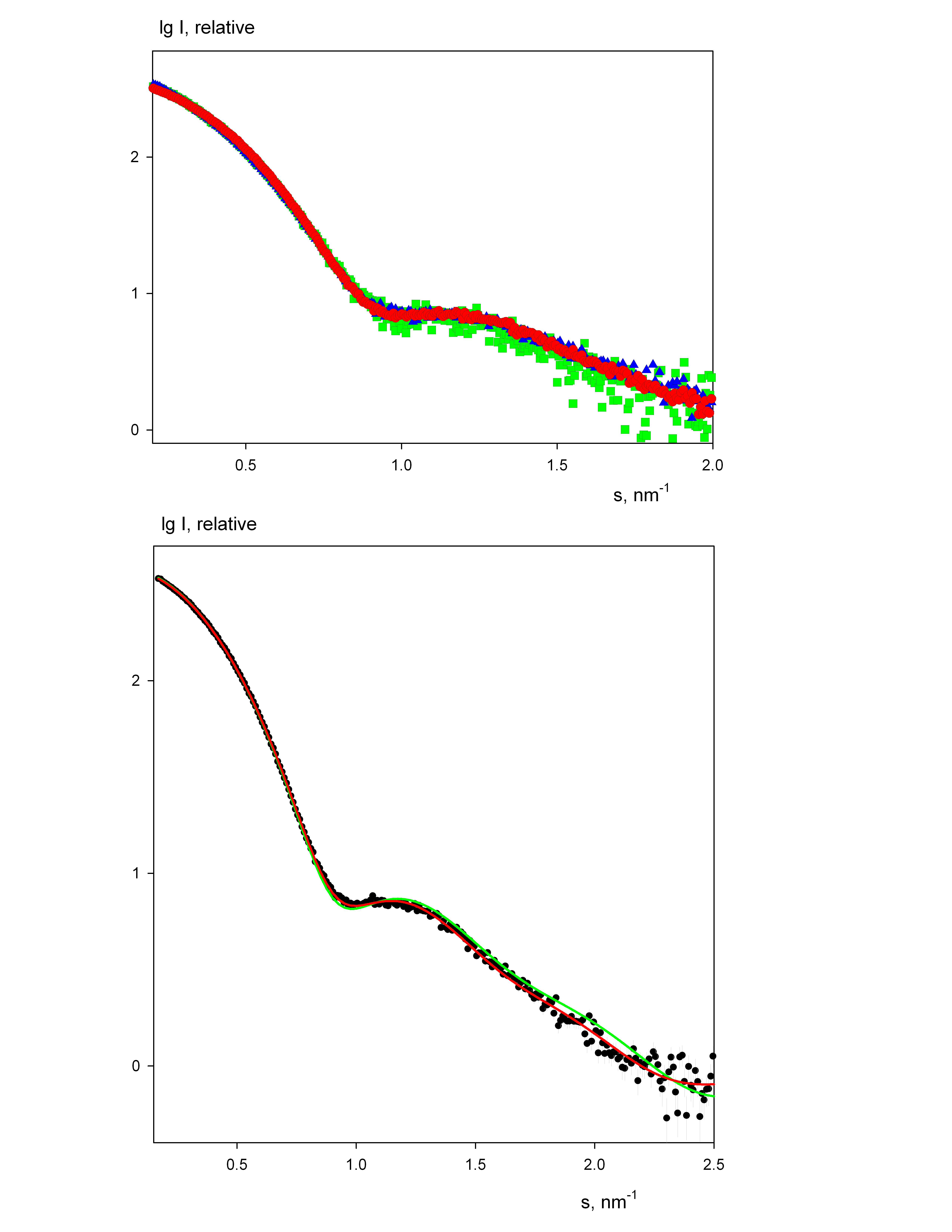


**Supplementary Figure S2.** SAXS scattering curves of RepB_6_ and calculated fits of X-ray structures. (**A**) Experimental scattering curves from concentration series of free RepB_6_ in solution. The data collected at 2.9 mg/ml, 5.8 mg/ml and 12.1 mg/ml are shown as green, blue and red dots, respectively. (**B**) Fits calculated from a mixture of C2 and C3 crystal structures (green) and from 12 MD snapshots selected by EOM (red) to the experimental data at 12.1 mg/ml (black dots).

**A**

**B**

**A**


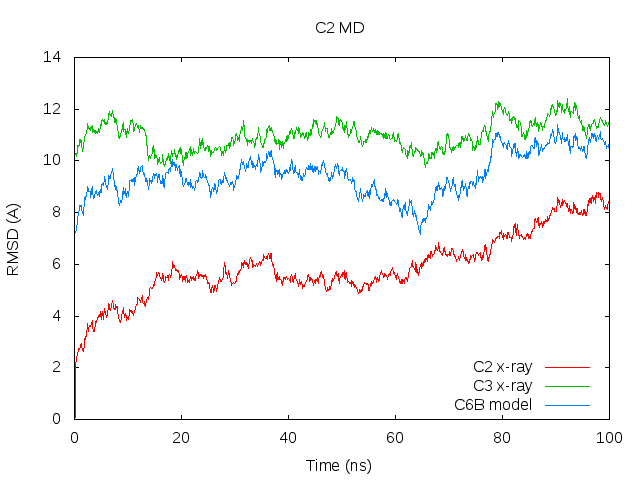

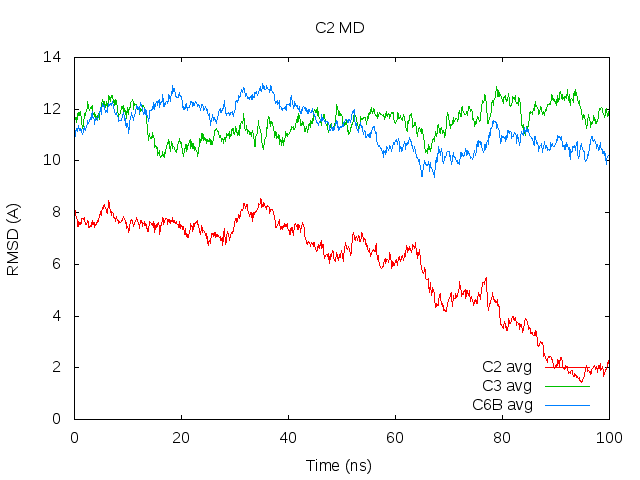


**B**


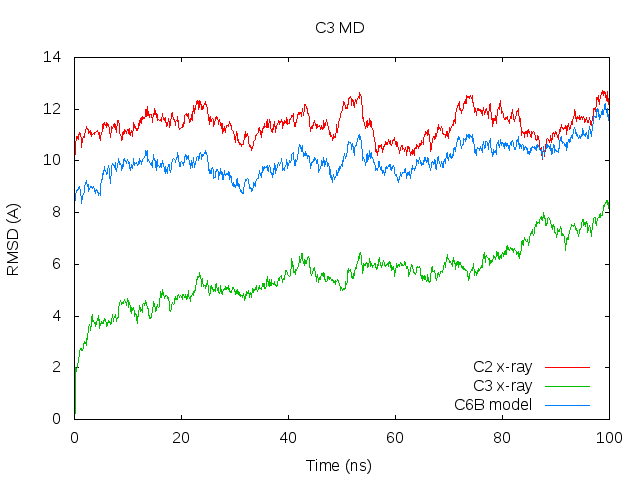

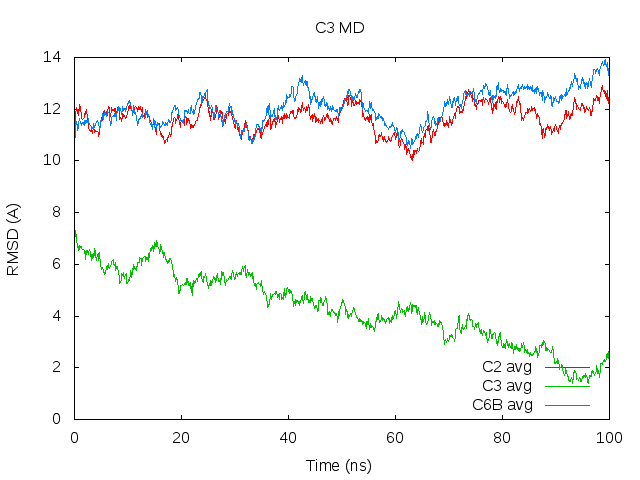


**C**


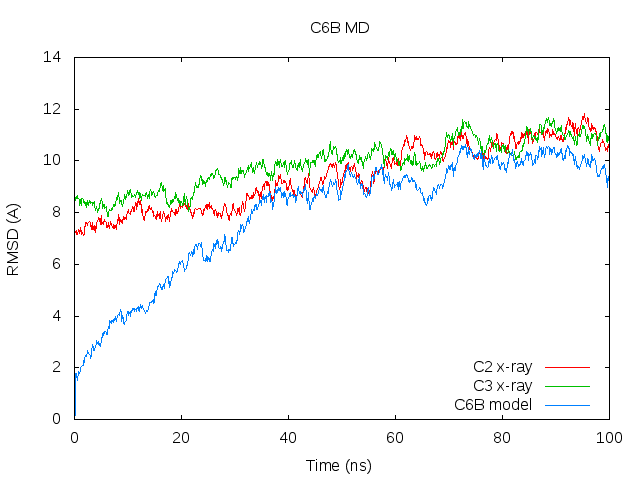

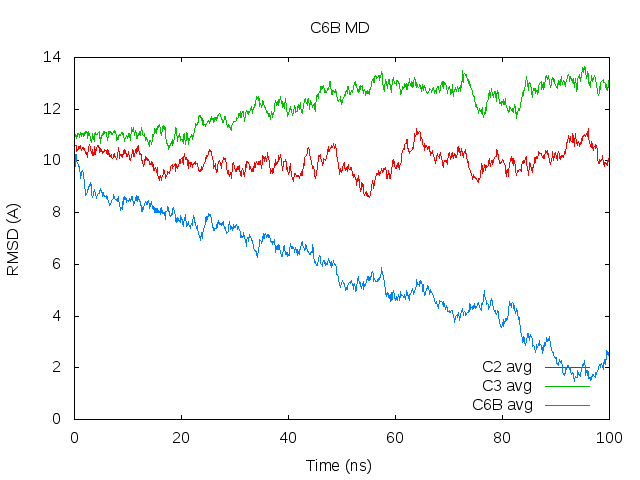


**Supplementary Figure S3.** C_α_ RMSD values with respect initial (top) and average structures from the last 10 nanoseconds (bottom) during 100 nanoseconds MD simulations started from the structures: (**A**) C2 (PDB ID: 3DKX), (**B**) C3 (PDB ID: 3DKY), and (**C**) C6B (model, see main text). We are reporting the minimum RMSD value after performing a rotational symmetry search with the six possible chain permutations.

**A**


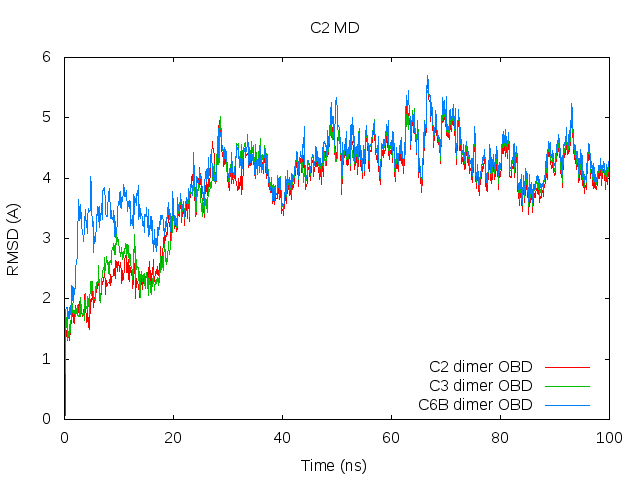


**B**


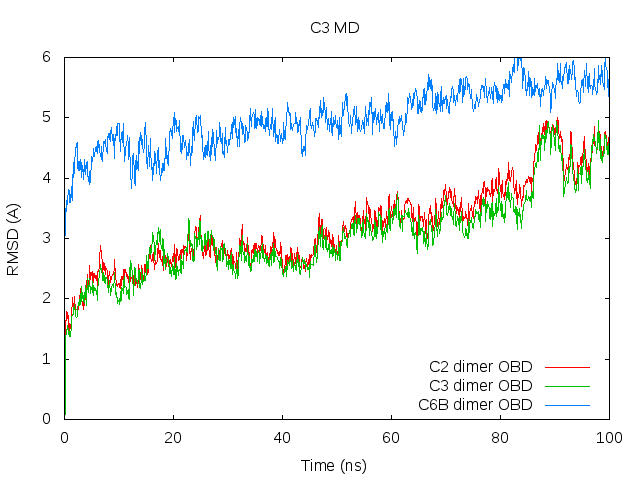


**C**

**
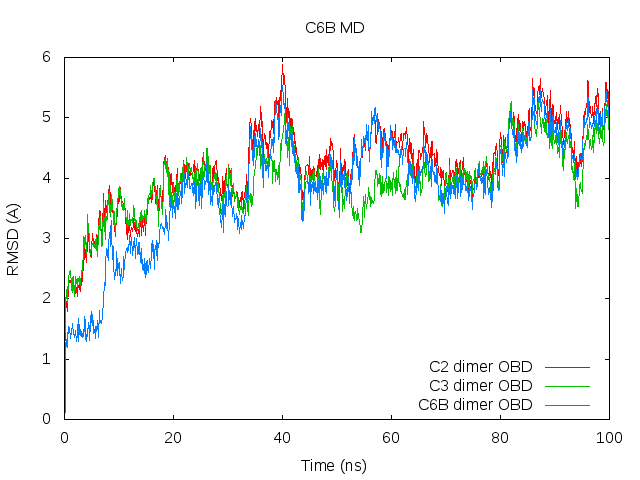
**

**Supplementary Figure S4.** Minimum Cα RMSD for a dimeric OBD domain during 100 nanoseconds MD simulations started from the structures: (**A**) C2 (PDB ID: 3DKX), (**B**) C3 (PDB ID: 3DKY), and (**C**) C6B (model). We are reporting the minimum RMSD value after performing an exhaustive rotational symmetry search with all the possible dimer permutations.

**A**


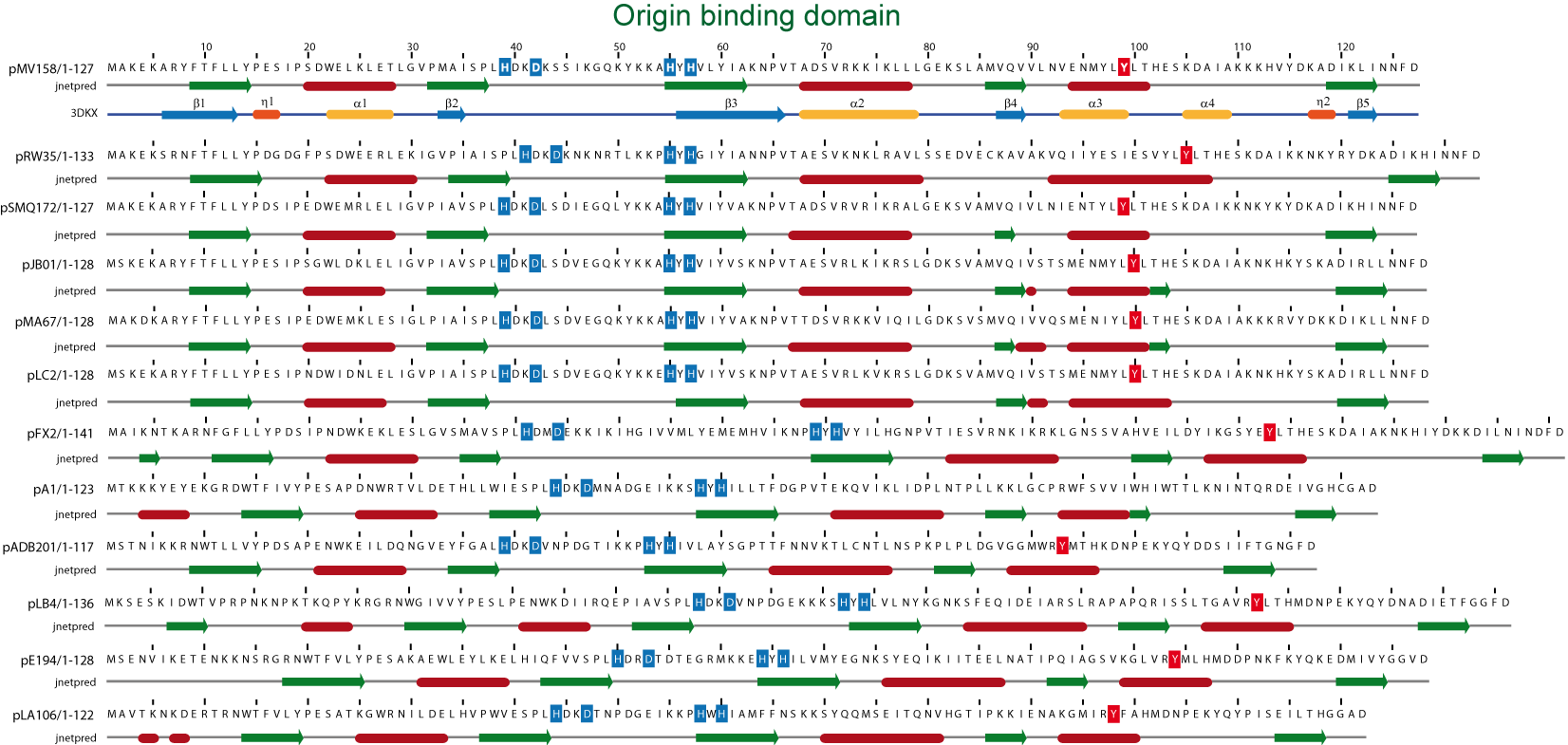


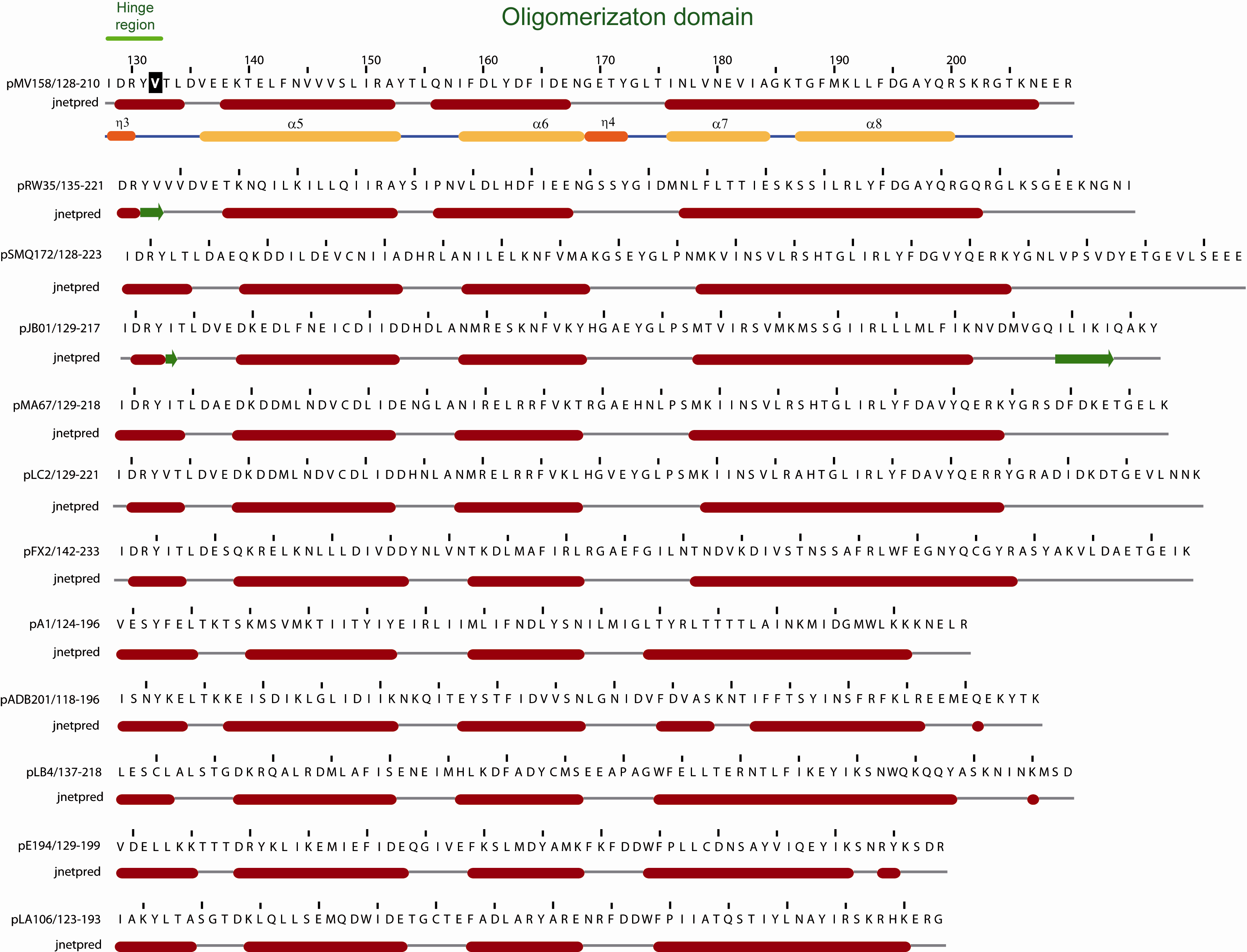
**B**

**Supplementary Figure S5.** Secondary structure predictions of the replication proteins of different rolling circle replication plasmids of the pMV158 family. The sequences of the N-terminal region (panel **A**) and the C-terminal region (panel **B**) and the corresponding secondary structure prediction are shown (the predicted α-helices and β-strands are represented as red bars and green arrows respectively). Conserved amino acid residues of the active site involved in metal binding and in the endonucleolytic activity are indicated by blue and red boxes, respectively, in the sequences. The additional line below the sequence of RepB shows the secondary structure present in the crystal structure of the protein (PDB entry code is given in the figure).


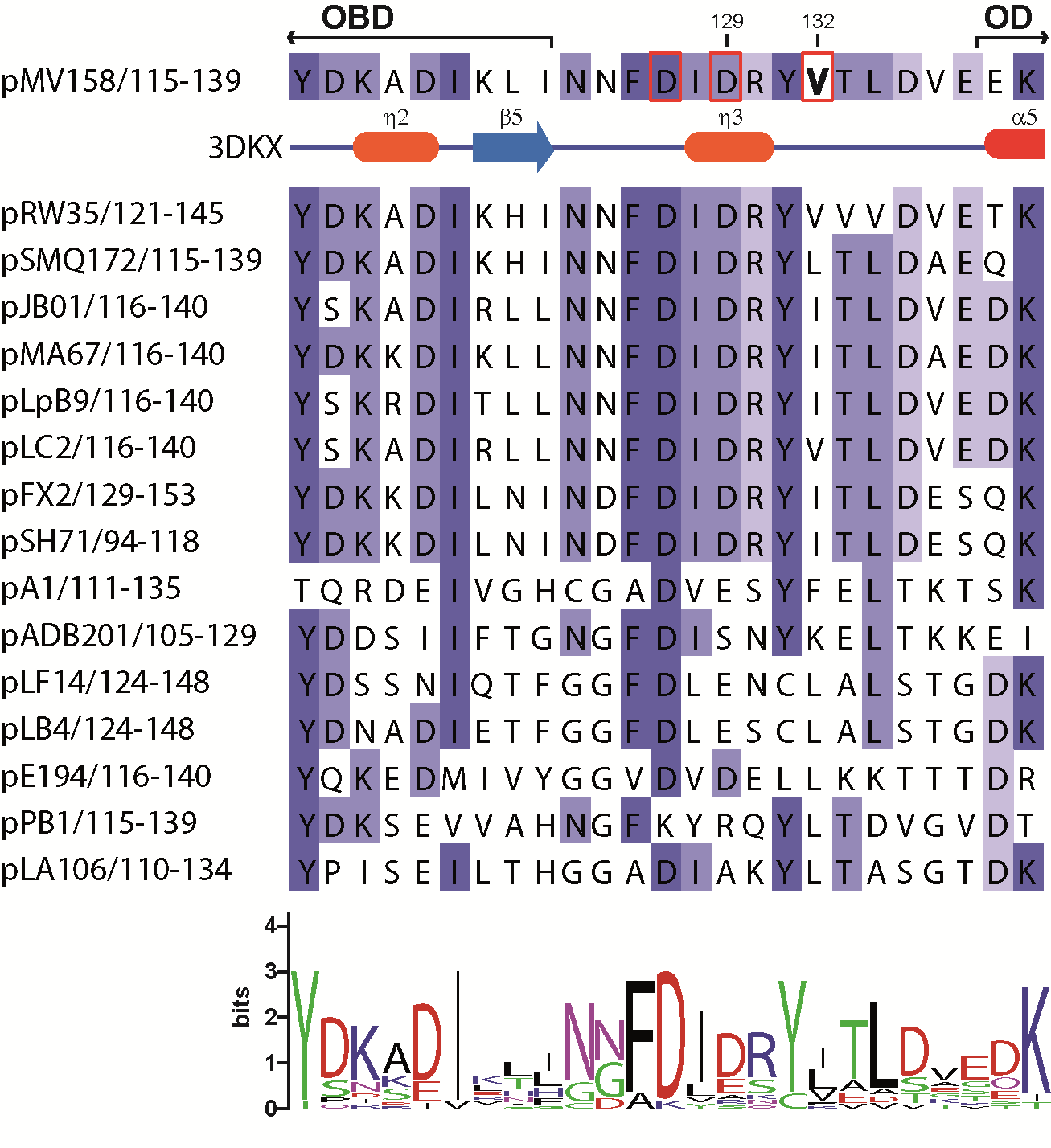


**Supplementary Figure S6.** Sequence alignment of the putative interdomain hinge region of initiator proteins of the pMV158 family. The residues D129 and V132 of the hinge region of RepB are highlighted in red. The secondary structure of RepB in the crystal structures is indicated below the amino acid sequence of the protein (PDB entry code is given). Boundaries of the RepB-OBD and OD domains are also indicated. The protein residue numbers of the interval showed in the alignment are indicated together with the plasmid name. Only the residues identical to the consensus sequence are coloured with different intensity depending on the degree of conservation; dark blue >80%, blue >60%, light blue >40%. Weblogo ^1^ was used to generate a logo with the sequences included in the alignment. The height of each letter is proportional to the frequency of the corresponding amino acid whereas the overall height of each stack of letters is proportional to the sequence conservation, measured in bits, at that position.

**References.**

1 Crooks, G. E., Hon, G., Chandonia, J.-M. & Brenner, S. E. WebLogo: A Sequence Logo Generator*. Genome Resear*c**h** 14, 1188-1190, doi:10.1101/gr.849004 (2004).
